# Life and Death of Immature Neurons in the Juvenile and Adult Primate Amygdala

**DOI:** 10.3390/ijms22136691

**Published:** 2021-06-22

**Authors:** Loïc J. Chareyron, Pamela Banta Lavenex, David G. Amaral, Pierre Lavenex

**Affiliations:** 1 Cognitive Neuroscience and Neuropsychiatry, University College London Great Ormond Street Institute of Child Health, 30 Guilford Street, London WC1N 1EH, UK; 2 Faculty of Psychology, UniDistance Suisse, 3900 Brig, Switzerland; pamela.banta@unidistance.ch; 3 California National Primate Research Center, Department of Psychiatry and Behavioral Sciences, MIND Institute, University of California, Davis, CA 956616, USA; dgamaral@ucdavis.edu; 4 Laboratory of Brain and Cognitive Development, Institute of Psychology, University of Lausanne, 1015 Lausanne, Switzerland; pierre.lavenex@unil.ch

**Keywords:** amygdala, primate, neuroblast, subventricular zone, lesion, hippocampus, neurodevelopmental disorders, Williams syndrome

## Abstract

In recent years, a large population of immature neurons has been documented in the paralaminar nucleus of the primate amygdala. A substantial fraction of these immature neurons differentiate into mature neurons during postnatal development or following selective lesion of the hippocampus. Notwithstanding a growing number of studies on the origin and fate of these immature neurons, fundamental questions about the life and death of these neurons remain. Here, we briefly summarize what is currently known about the immature neurons present in the primate ventral amygdala during development and in adulthood, as well as following selective hippocampal lesions. We provide evidence confirming that the distribution of immature neurons extends to the anterior portions of the entorhinal cortex and layer II of the perirhinal cortex. We also provide novel arguments derived from stereological estimates of the number of mature and immature neurons, which support the view that the migration of immature neurons from the lateral ventricle accompanies neuronal maturation in the primate amygdala at all ages. Finally, we propose and discuss the hypothesis that increased migration and maturation of neurons in the amygdala following hippocampal dysfunction may be linked to behavioral alterations associated with certain neurodevelopmental disorders.

## 1. Introduction

### 1.1. A Brief History of the Study of Immature Neurons in the Primate Amygdala

Although the paralaminar nucleus of the amygdala is obviously more darkly stained in Nissl preparations than other amygdala nuclei, Bernier and Parent [1,2] were the first to report the presence of small immature neurons immunoreactive for the anti-apoptosis proto-oncogene Bcl-2 in the basal portion of the monkey amygdala. Shortly after, the same population of immature neurons was identified in the human amygdala by Yachnis et al. [3], who noted that this population of Bcl-2-positive cells is most abundant during infancy but gradually decreases throughout adulthood. Bernier and colleagues [4] further described the migration of BrdU-positive newly generated neurons from the subventricular zone (SVZ) at the tip of the temporal horn of the lateral ventricle (tLV) into the paralaminar nucleus of adult monkeys, thus proposing an origin for these adult-generated neurons. These neurons are not only immunoreactive for Bcl-2, but also for PSA-NCAM, doublecortin (DCX) and β-tubulin-III [4,5,6], all markers of immature neurons. Zhang and colleagues [7] reported that the immature neurons in the ventral amygdala co-express the neuron-specific nuclear protein (NeuN) and DCX, and that although the number of these cells decreases in the paralaminar nucleus across the lifespan, a significant population is nonetheless present in 12-year-old, 21-year-old, and even 31-year-old monkeys. In 2012, the first comprehensive review on the primate paralaminar nucleus was published by deCampo and Fudge [8] and interest in this nucleus has been increasing ever since.

**Figure 1:**
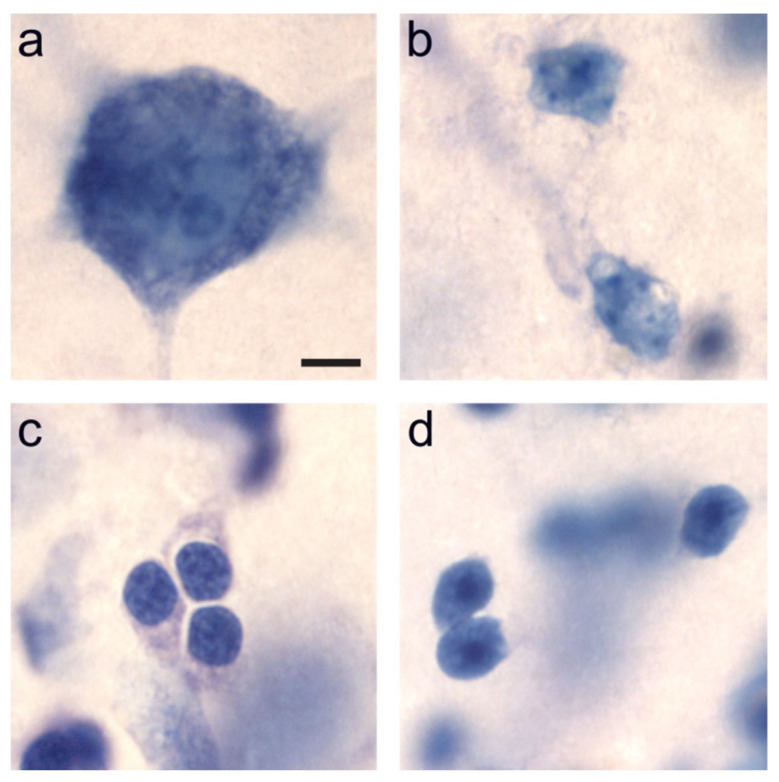
Classification and identification of different cell types in the monkey amygdala, viewed with a ×100 objective in Nissl-stained, coronal sections cut at 60 µM. (**a**) Neuron; (**b**) Astrocytes; (**c**) Oligodendrocytes; (**d**) Immature neurons. We distinguished mature neurons, astrocytes, oligodendrocytes, and immature neurons based on morphological criteria identifiable in Nissl preparations [9,10,13,14]. We refer the reader to the original publications [15,16,17,18] for detailed descriptions. Briefly, neurons are darkly stained and comprise a single large nucleolus. Astrocytes are relatively smaller in size and exhibit pale staining of the nucleus. Oligodendrocytes are smaller than astrocytes and contain round, darkly stained nuclei that are densely packed with chromatin. Immature neurons are small with round to slightly oval, hyperchromatic nuclei containing distinguishable nucleoli [3,4,5]. Scale bar in (**a**) = 5 µM (applies to all). Reproduced from [9].

Our research group was the first to quantify this population of immature neurons in the monkey amygdala at different ages during early postnatal life and in adulthood [9]. Using design-based stereological techniques on Nissl-stained sections (Figure 1), we found that the number of immature neurons present in the monkey paralaminar nucleus remains stable across the first year of life (at approximately 1 million neurons) and then decreases sharply between one and five years of age. The decrease in the number of immature neurons is paralleled by an increase in the number of mature neurons in the paralaminar nucleus, suggesting that neuronal differentiation occurs in the amygdala during a period corresponding to childhood and adolescence in primates. We also found a large and persistent population of about 500,000 immature neurons in the paralaminar nucleus of 5–9-year-old monkeys [9]. In contrast, we found neither the presence of a comparable population of Bcl-2-positive immature-looking neurons nor any change in the number of mature neurons in the rat amygdala during postnatal development [10], suggesting that this late and relatively massive maturation of neurons in the ventral amygdala may be restricted to non-rodent mammalian species [11]. This observation, however, does not preclude the possible presence of isolated newly generated neurons in the rodent amygdala [12].

Sorrells et al. [19] confirmed some of these findings in humans. Although they did not quantify the number of cells, they reported that a portion of the population of small DCX-positive cells found in the ventral amygdala mature into large DCX-negative neurons during childhood. The analysis of neurogenesis or immature neuron markers, like DCX, in human immersion-fixed tissue samples is challenging and the comparison between observations made in different species should be interpreted carefully [20,21,22,23]. Interestingly, Sorrells et al. performed a detailed genetic analysis of paralaminar neurons using RNA sequencing and identified a highly distinct cluster of cells expressing DCX, Bcl-2, as well as other markers of immature neurons [19]. By analyzing cell densities over time, they concluded that immature neurons in the paralaminar nucleus slowly mature and that this process is most pronounced during adolescence.

### 1.2. Regulation of Immature Neurons in the Primate Amygdala

Recognizing the spatial proximity between the population of immature neurons in the ventral amygdala and where projections from the anterior hippocampus terminate in the amygdala, Fudge and colleagues were the first to propose a potential functional link between the maturation of neurons in the ventral amygdala and neurogenesis in the dentate gyrus [5,24]. In order to empirically investigate whether hippocampus (dys)function may influence the process of neuronal maturation in the ventral amygdala, we set out to document the impact of neonatal or adult lesions of the hippocampus on different neuronal populations in the monkey amygdala [13]. In adult monkeys that had received selective hippocampus lesions in adulthood, we found an increase in the number of mature neurons and a concomitant decrease in the number of immature neurons in the paralaminar nucleus. In contrast, in adult monkeys that had received selective hippocampus lesions shortly after birth, we observed a massive increase in the number of mature neurons in the paralaminar, lateral and basal nuclei of the adult amygdala, as well as a massive increase in the population of immature neurons in the paralaminar nucleus. These findings thus suggested that whereas adult hippocampus lesions lead only to an increase in the differentiation of immature neurons in the paralaminar nucleus, neonatal hippocampus lesions lead to an increase in the differentiation of immature neurons in the paralaminar nucleus and neighboring amygdala nuclei, and the concomitant migration of neuroblasts from the SVZ to the paralaminar nucleus.

Given the known role of the amygdala in emotional and social behavior, Marlatt et al. [6] evaluated the impact of exposure to 2 weeks of isolation and social defeat stress on the number of DCX-positive immature neurons and BrdU-positive cells in the adult marmoset amygdala. Although they did not find any difference in the populations of BrdU-positive and DCX-positive cells between marmosets subjected to stress and controls following a 2-week recovery period, their study nevertheless confirmed the presence of a large population of immature neurons in the ventral amygdala in another non-human primate species. Their study also questioned whether the maintenance of such a population of immature neurons in the primate amygdala may be dependent on the migration of neuroblasts from the SVZ.

In humans, Avino et al. [25] analyzed the number of neurons in the main amygdala nuclei in post-mortem tissue from neurotypical individuals (control) and individuals with autism spectrum disorder (ASD). For control individuals, they found that the number of mature neurons increases in the basal nucleus from childhood to adulthood, coinciding with a decrease of immature neurons in the paralaminar nucleus (NB: in their study the paralaminar nucleus was included as part of the basal nucleus). For individuals with ASD, in contrast, neuron number is greater than in controls in the basal and central nuclei during childhood, but lower than in controls in the lateral, basal, and accessory basal nuclei in adulthood, suggesting an altered maturation profile of these neurons across the lifespan in ASD. In another study, Sorrells et al. [19] compared gene expression in the immature amygdala cell population in control and ASD brains and identified differences in expression of a small number of ASD-associated genes, many of which are essential for cell adhesion and cytoskeleton function.

Altogether, these studies provide consistent evidence that immature neurons present in the paralaminar nucleus of the primate amygdala differentiate into mature neurons over the course of normal postnatal development, and that the maturation of these neurons may be impacted by experimental, genetic, or epigenetic factors. What remains to be determined is how late-maturing neurons from the paralaminar nucleus integrate into established amygdala circuits and what their function may be. The delayed maturation of these neurons is likely to have a prominent functional impact as these late maturing neurons represent almost 10% of the total number of mature neurons present in the basolateral portion of the amygdala (i.e., including the lateral, basal and paralaminar nuclei) in adult monkeys [9]. Moreover, whereas evidence of neuronal migration from the SVZ of the temporal horn of the lateral ventricle to the amygdala has been reported in adult monkeys [4], the long-term fate of these neurons has yet to be fully characterized.

Here, we report new observations on the distribution of immature neurons in the adult monkey medial temporal lobe alongside a more detailed analysis of some of our previous stereological investigations in postnatally developing and adult monkeys [9,13,14]. These data provide further evidence suggesting that neuroblast migration from the SVZ likely occurs in parallel with the maturation of immature neurons in the paralaminar nucleus during normal adolescence, as well as following selective hippocampus lesion. Finally, we propose the hypothesis that enhanced migration to and maturation of neurons in the amygdala following hippocampus dysfunction may lead to some functional abnormalities observed in some neurodevelopmental disorders.

## 2. Results

### 2.1. Distribution of Immature Neurons in the Primate Medial Temporal Lobe

**Figure 2:**
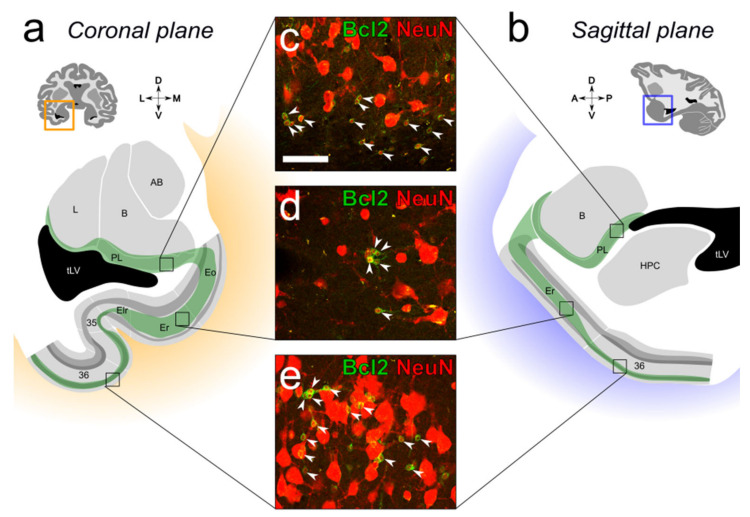
Distribution of Bcl2+/NeuN+ immature neurons in the medial temporal lobe of the adult monkey. The distributions of Bcl2+/NeuN+ immature neurons are represented as green areas on typical coronal (**a**) and sagittal (**b**) sections; (**c**) The largest population of Bcl2+/NeuN+ immature neurons is found in the medial and caudal parts of the paralaminar nucleus lying next to the temporal lobe lateral ventricle; (**d**) Bcl2+/NeuN+ immature neurons are found in the anterior portion of the entorhinal cortex (areas Eo, Er and Elr); (**e**) A large number of Bcl2+/NeuN+ immature neurons is also observed in layer II of the perirhinal cortex (areas 35 and 36). Abbreviations: B, basal nucleus; L, lateral nucleus; AB, accessory basal nucleus; HPC, hippocampus; PL, paralaminar nucleus; tLV, temporal lobe lateral ventricle. Scale bar in (**c**) = 50 µM, applies to panels (**d**,**e**).

In monkeys, immature NeuN-positive neurons expressing the anti-apoptosis Bcl-2 marker are present in the ventral amygdala (mainly in the paralaminar nucleus), in several layers of the anterior portion of the entorhinal cortex, and form a particularly high-density population in layer II of the perirhinal cortex (areas 35 and 36) (Figure 2).

### 2.2. Immature Neurons from Birth to One Year of Age in Monkeys

**Figure 3:**
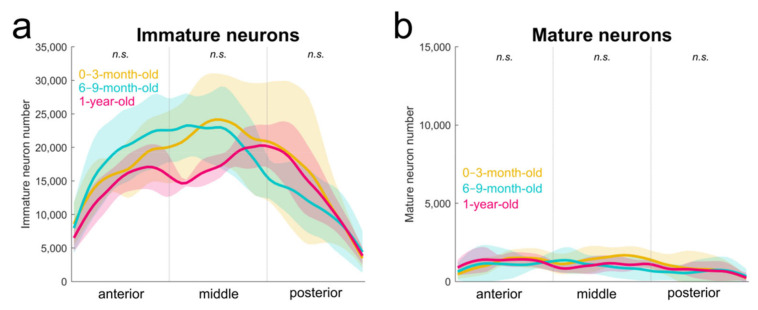
Distributions of immature (**a**) and mature (**b**) neurons in the paralaminar nucleus of the amygdala in 0–3-month-old (*n* = 8; orange), 6–9-month-old (*n* = 8; blue), and 1-year-old (*n* = 4; red) control monkeys (average ± SD). Based on neuron number estimates reported in [9]. n.s., no statistically significant difference.

In order to characterize the normal structural development of the amygdala in the macaque monkey, we previously used design-based stereological methods to estimate the number of neurons in the main amygdala nuclei at different postnatal ages, from birth to young adulthood [9]. Here, we performed a more detailed analysis of these estimates to characterize the anteroposterior distribution of these neurons. We confirmed the absence of differences in the number of mature or immature neurons or in the total number of neurons (mature + immature) in the paralaminar nucleus between 0–3-month-old, 6–9-month-old and 1-year-old monkeys (1-way ANOVAs, all F_(2,17)_ < 0.78, all *p* > 0.47). Importantly, we did not find any differences in the distribution of immature or mature neurons along the anteroposterior axis of the paralaminar nucleus within the first year of life (Figure 3; Table 1; 2-way ANOVA interaction, all F_(4,51)_ < 1.4, all *p* > 0.25).

### 2.3. Immature Neurons from One Year to 5–9 Years of Age in Monkeys

**Figure 4:**
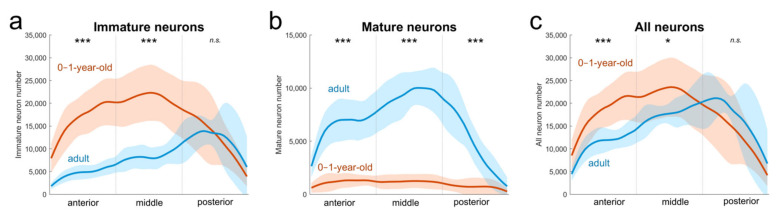
Distribution of immature (**a**), mature (**b**), and all (mature + immature) (**c**) neurons in the paralaminar nucleus of the amygdala in juvenile (< 1 year; *n* = 20; red), and adult (>5 years; *n* = 7; blue) control monkeys (average ± SD; *** *p* < 0.001; * *p* < 0.05; n.s., no statistically significant difference). Based on neuron number estimates reported in [9,13].

We have previously shown that the number of immature neurons decreases in the paralaminar nucleus between one year and 5–9 years of age in monkeys [9] (Table S1). However, our new analyses indicate that this decrease is not uniform along the anteroposterior extent of the paralaminar nucleus (Table 1; Figure 4a; 2-way ANOVA interaction, F_(2,75)_ = 14.1, *p* < 0.001). There are fewer immature neurons in the anterior and middle thirds of the paralaminar nucleus in adult (5−9 years of age) than in juvenile (0−1 year of age) monkeys (Holm-Sidak test: both *p* < 0.001), but no difference in the number of immature neurons in the posterior third (*p* = 0.71). This observation may be interpreted to suggest that the immature neurons located in the posterior part of the paralaminar nucleus do not mature over the course of normal postnatal development. However, such interpretation is not consistent with the finding that the number of mature neurons in the posterior third of the paralaminar nucleus increases by 80,000, a 550% increase, after one year of age (2-way ANOVA interaction, F_(2,75)_ = 36.25, *p* < 0.001; Holm–Sidak test: *p* < 0.001; Figure 4b), thus suggesting instead that neuronal maturation is indeed occurring in this part of the nucleus. Accordingly, it is possible that the large number of immature neurons found in the posterior third of the paralaminar nucleus in adult monkeys has actually migrated from the caudally situated SVZ to the paralaminar nucleus after one year of age.

In support of this interpretation, our new analyses revealed that the total number of neurons (mature + immature) is slightly higher in the posterior paralaminar nucleus of an adult than juvenile monkeys (+25%, even though the difference was not statistically significant, 2-way ANOVA interaction, F_(2,75)_ = 7.76, *p* < 0.001; Holm–Sidak test, *p* = 0.082; Figure 4c). In contrast, the total number of neurons in the anterior and middle thirds of the paralaminar nucleus was lower in adult than in juvenile monkeys (−38% and −20% respectively, both *p* < 0.021).

### 2.4. Neuronal Migration Following Selective Lesion of the Hippocampus

We previously studied the effects of selective adult or neonatal hippocampus lesions on the populations of mature and immature neurons in the main nuclei of the adult monkey amygdala [13]. The monkeys with adult hippocampus lesions received bilateral ibotenic acid lesions of the hippocampus when they were 6.7–9.7 years of age and were killed 42 months post-lesion [26]. The monkeys with neonatal hippocampus lesion received bilateral ibotenic acid lesions of the hippocampus at 12–16 days after birth and were killed when they were 9.1−9.4 years of age [27]. The number of neurons in the amygdala was determined using design-based stereological techniques. Compared with unoperated controls, the number of mature neurons was about 70% higher in the paralaminar nucleus of adult- and neonate-lesioned monkeys, and 40% higher in the lateral and basal nuclei of neonate-lesioned monkeys. The number of immature neurons in the paralaminar nucleus was 40% higher in neonate-lesioned monkeys and 30% lower in adult-lesioned monkeys. To confirm our estimates of the number of immature neurons derived from Nissl-stained preparations, we performed a stereological analysis of the number of Bcl2-positive cells. The estimates of the number of immature neurons in Nissl-stained sections and the number of Bcl2-positive cells were highly correlated (Pearson’s correlation, r = 0.83, *p* < 0.001). Moreover, the anteroposterior distributions of immature neurons defined from Nissl-stained and Bcl2-immunolabeled preparations were very similar, and the differences in the number of immature neurons between different experimental groups were essentially the same with both techniques (Figure S1). Finally, the total number of neurons (mature + immature) in the paralaminar nucleus was 53% larger in neonate-lesioned monkeys than in controls (*p* < 0.001), and 14% larger in adult-lesioned monkeys than in controls (*p* = 0.042) [13]. These increases in total neuron numbers, therefore, suggested that migration of immature neurons to the amygdala occurred following neonate and adult hippocampus lesion.

Although the increased number of mature neurons in the paralaminar nucleus of the late- and early-lesioned groups was accompanied by an enlargement of the paralaminar nucleus as compared to controls (+27% and +26% respectively, F_(2,18)_ = 15.6; *p* < 0.001; Fisher’s LSD, both *p* < 0.001; Table S1), the density of mature neurons was also greater in these two groups than in controls (+29% and +36% respectively; F_(2,18)_ = 25.14; *p* < 0.001; Fisher’s LSD, both *p* < 0.001). In contrast, although the number of mature neurons was 40% higher in the lateral and basal nuclei of early-lesioned monkeys, the volumes of these nuclei were not different from control or late-lesioned groups (lateral, F_(2,18)_ = 0.60; *p* = 0.56; basal, F_(2,18)_ = 0.89; *p* = 0.43). As a consequence, the density of mature neurons was 30% and 20% greater than in controls in the lateral and basal nuclei respectively (lateral, F_(2,18)_ = 8.6; *p* = 0.002; basal, F_(2,18)_ = 6.4; *p* = 0.008; Fisher’s LSD, both *p* < 0.026). Thus, following lesion of the hippocampus, the maturation of neurons in the amygdala is accompanied by important changes in its structural and cellular organization.

## 3. Discussion

The migration of newly generated neurons from the SVZ of the temporal horn of the lateral ventricle to the paralaminar nucleus in adult monkeys was first reported by Bernier and colleagues [4], but their number was not quantified and their fate after a few weeks was not evaluated. Here, we presented new findings indicating that the maturation of neurons in the monkey amygdala is likely paralleled by the migration of immature neurons from the lateral ventricle at all ages.

### 3.1. Distribution of Immature Neurons in the Primate Medial Temporal Lobe

Our current findings demonstrate that the population of immature neurons found in the ventral amygdala is not an isolated population but may be part of a larger group of Bcl-2-positive and NeuN-positive cells that extends from the SVZ of the lateral ventricle to the paralaminar nucleus and some cortical areas within the medial temporal lobe. The presence of these immature neurons in the ventral amygdala, anterior entorhinal cortex and layer II of the perirhinal cortex can be observed on coronal or sagittal sections of the temporal lobe labeled with DCX or Bcl-2 markers [4,5,7]. While the population of DCX-positive immature neurons found in layer II of several other cortical areas diminishes with age [7], it appears relatively constant in the amygdala, and in the entorhinal and perirhinal cortices.

### 3.2. Neuronal Migration during Development

Our findings suggest that substantial neuronal migration occurs during normal postnatal development in the monkey amygdala. During the first year of life, immature neurons in the monkey paralaminar nucleus remain in a protracted state of arrested maturation, maintaining a small size, simple morphology, and a persistent expression of immature markers, as was also shown in humans [19]. Interestingly, between one year and 5–9 years of age, the number of immature neurons decreases in the monkey paralaminar nucleus [9], but this decrease is not uniform along the anteroposterior extent of the nucleus. Indeed, our stereological estimates have shown that there are about 270,000 more mature neurons and about 490,000 fewer immature neurons in the anterior and middle thirds of the paralaminar nucleus in adult than in juvenile monkeys. Thus, about 45% (220,000) of the immature neurons present in the anterior and middle thirds of the paralaminar nucleus in juvenile monkeys are no longer present in adulthood (i.e., they are no longer present in the immature pool of neurons and they do not appear to have differentiated into mature neurons). One obvious hypothesis explaining the difference in the total number of neurons in juvenile and adult monkeys is that many immature neurons may die over the course of normal postnatal development. Indeed, programmed cell death (i.e., apoptosis) affects nearly all classes of neurons and it is generally assumed that about half of all neurons die during neural development [28,29,30]. Quantitatively, the vast majority of neuronal apoptosis occurs during early brain development, when neurons are in a relatively late stage of maturation and begin contacting the cells they innervate [31,32]. However, apoptosis has also been shown to occur within the neurogenic zones of the adult brain. For example, about half of adult-born neurons die shortly after their arrival in the olfactory bulb in mice [33]. A similar proportion of newly generated neurons has been shown to die in an adult canary vocal center [34]. In the dentate gyrus of the adult rodent hippocampal formation, 50–70% of the newly generated neurons degenerate within one month after their production [35,36]. Here, we used indirect but reliable measures of cell death based on design-based stereological estimates of neuron numbers. Direct measures of apoptosis (detecting DNA fragmentation) are limited by the relatively rare presence of apoptotic cells, since cellular degeneration has been reported to be very rapid, lasting just 72 h in the rat hippocampus [37], for example. Similarly, in primates, the numbers of apoptotic cells detected in the hippocampus have been extremely low [38,39,40,41], although neural cell death is also likely to parallel neuron production in the developing and adult dentate gyrus [38].

Another possible explanation for the decrease in the total number of neurons in the anterior and mid portions of the paralaminar nucleus between adolescence and adulthood might be that many immature neurons migrate into adjacent amygdala nuclei. The presence of PSA-NCAM expression in immature neurons of the primate paralaminar nucleus supports the idea that these neurons may be migrating [8,42]. In contrast, the random orientation of elongated immature neurons suggests that immature neurons are not migrating away from the paralaminar nucleus but are rather adjusting their final position [19]. Accordingly, we did not find any evidence of the approximately 220,000 missing neurons in either the adjacent lateral or basal nuclei, where the number of mature neurons is stable throughout postnatal development and where the presence of immature neurons across development remains only anecdotal [9].

**Figure 5:**
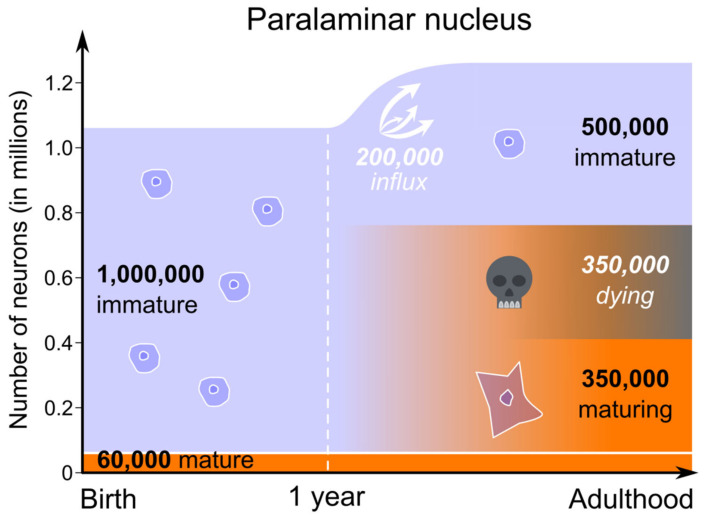
Proposed sequence of neuron maturation in the paralaminar nucleus of the monkey amygdala. Light blue: immature neuron population; orange: mature neuron population. Numbers in white italic are speculative (number of dying and migrating cells). The number of dying neurons is based on the hypothesis that ~50% of neurons die in the course of maturation (see main text). All other neuron number estimates were obtained with design-based stereological techniques applied to Nissl-stained brain sections of monkeys at different postnatal ages (*n* = 27; [9,13]).

In sum, our calculations suggest that between one year and adulthood, approximately 350,000 immature neurons (from a pool of approximately 1 million immature neurons present at birth) mature in the paralaminar nucleus of the monkey amygdala (270,000 in the anterior and middle third and 80,000 in the posterior third). Since a variety of observations suggest that about 50% of maturing neurons die over the course of normal development, this suggests that approximately 350,000 immature neurons also died during this period. However, because we observed 500,000 immature neurons in the adult paralaminar nucleus, it stands to reason that from one year of age to adulthood at least 200,000 immature neurons migrated from a region caudal to the amygdala into the paralaminar nucleus (Figure 5). This number represents about 40% of the number of immature neurons found in the paralaminar nucleus of adult monkeys.

It is also important to recognize that our inferred estimate of immature neuronal cell death in the developing amygdala may even be an underestimation. Indeed, our estimation is based on the assumption that there is no postnatal migration of immature neurons into the anterior and middle thirds of the paralaminar nucleus, and thus that the decrease in total neuron number in these portions of the nucleus is explained solely by the maturation and death of immature neurons already present in these regions at birth. Our experimental design did not allow us to determine if an influx of immature neurons may also parallel the maturation of a similar number of immature neurons in the anterior and middle thirds of the paralaminar nucleus. If this is found to be the case, the level of immature neuronal cell death could be even greater than the estimated 50%.

### 3.3. Neuronal Migration Following Selective Lesion of the Hippocampus

Our recent studies revealed that following lesion of the hippocampus there must be a substantial migration of immature neurons from the SVZ of the temporal horn of the lateral ventricle to the paralaminar nucleus of the amygdala in juvenile and adult monkeys [13]. Many of these neurons appear to survive for several years after the lesion, and a significant number of them become morphologically mature.

#### 3.3.1. Adult Hippocampus Lesion

**Figure 6:**
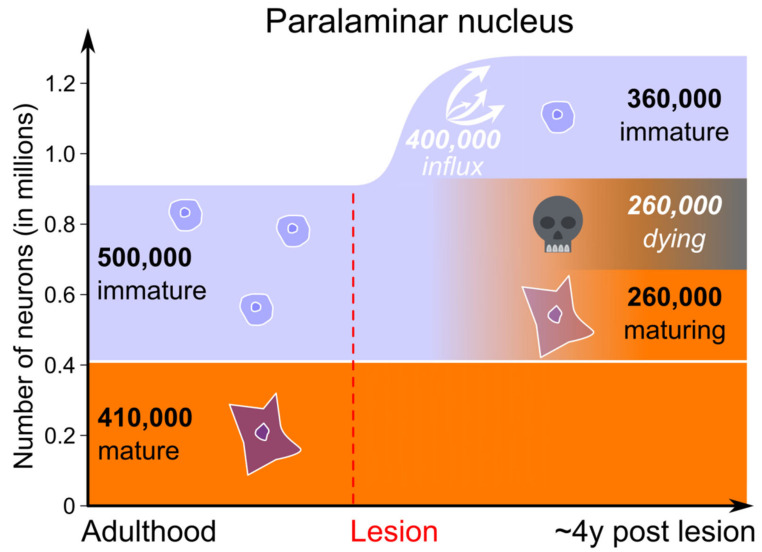
Proposed sequence of neuron maturation in the paralaminar nucleus of the monkey amygdala following adult hippocampal lesion. Light blue: immature neuron population; orange: mature neuron population. Numbers in white italic are speculative (number of dying and migrating cells). The number of dying cells is based on the hypothesis that ~50% of neurons die in the course of maturation (see main text). All the other cell number estimates were obtained with design-based stereological techniques applied to Nissl-stained monkey brain sections (Control monkeys *n* = 7; Lesioned monkeys *n* = 6; [9,13]).

As compared to controls, the number of mature neurons was 65% higher in the paralaminar nucleus of adult-lesioned monkeys, while the number of immature neurons was only 27% lower. Also, there were about 130,000 (14%) more total neurons (mature + immature) in the paralaminar nucleus of the adult-lesioned monkeys than in controls. Even if we assume that all paralaminar neurons that had completed the maturational process survived in hippocampus-lesioned monkeys, these observations strongly suggest that a relatively large population of immature neurons migrated to the paralaminar nucleus following selective hippocampus lesion in adulthood. However, because it is also reasonable to estimate that approximately 50% of the immature neurons found in the amygdala may have died after one year of age in monkeys (see above). We therefore hypothesize that an even larger number of immature neurons may have migrated from the lateral ventricle to the paralaminar nucleus following adult hippocampus lesion (Figure 6).

Increased SVZ proliferation and neurogenesis have been documented following traumatic brain injury, intracerebral hemorrhage or ischemia in rodents [43,44,45]. In primates, an increase in the number of progenitor cells in the SVZ is observed after ischemic injuries in adult monkeys [46,47,48]. In humans, increased numbers of neural cells with immature phenotypes have been reported in the SVZ of adult patients following traumatic brain injury or ischemic stroke [49,50,51]. While progenitor cell migration toward the damaged area has been observed in rodents [45,52,53,54], migrating progenitor cells observed in the adult primate brain following ischemia do not appear to deviate from their normal migratory pathway [47,50] or deviate for only very short distances [48]. This could be due to a more generally restricted migratory capacity of primate neurons [55]. If this is the case, immature neurons migrating toward the amygdala in hippocampus-lesioned monkeys may not be directed toward the damaged region but may instead be following an existing migratory stream [4,13].

#### 3.3.2. Neonatal Hippocampus Lesion

**Figure 7:**
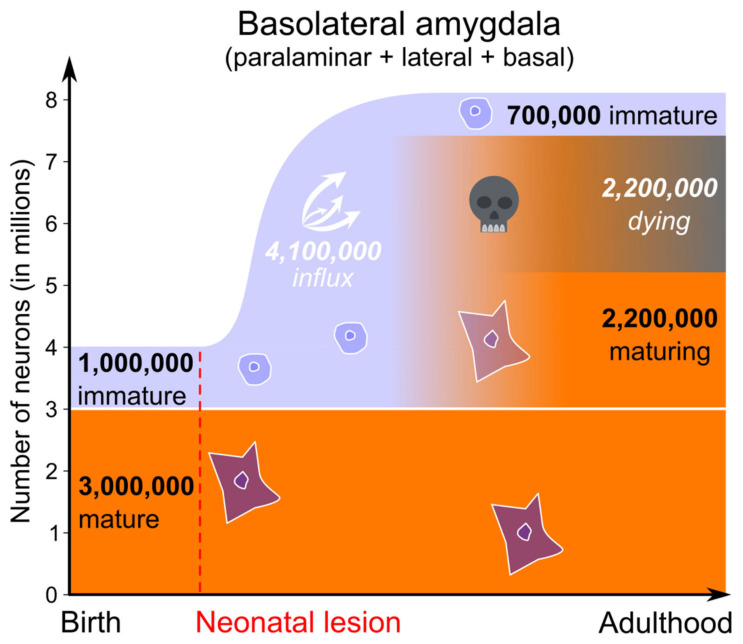
Proposed sequence of neuron maturation in the monkey basolateral amygdala (lateral, basal and paralaminar nuclei) following neonatal hippocampal lesion. Light blue: immature neuron population; orange: mature neuron population. The relatively low number of immature neurons present in the lateral and basal nuclei is not considered here. Numbers in white italic are speculative (number of dying and migrating cells). The number of dying cells is based on the hypothesis that ~50% of neurons die in the course of maturation (see main text). All the other cell number estimates were obtained with design-based stereological techniques applied to Nissl-stained monkey brain sections (Control monkeys *n* = 4; Neonatal-lesioned monkeys *n* = 8; [9,13]).

The total number of neurons in the basolateral amygdala (lateral + basal + paralaminar) of neonatal-lesioned monkeys was 46% higher than in juvenile controls (~1,900,000 more total neurons). Taking into account the likely cell death that accompanies neuronal maturation, we can thus estimate that the number of immature neurons migrating from the ventricle to the amygdala following a neonatal hippocampus lesion may be about ten times higher than following adult lesion (i.e., 4,100,000 vs. 400,000 neurons) (Figure 7).

Although neonate-lesioned monkeys lived longer (2.5 × times longer after early lesion (9 years) than after late lesion (3.5 years)) before being sacrificed than adult-lesioned monkeys, post-lesion survival time is alone unlikely to explain the difference in the number of neurons estimated to have migrated to the amygdala in early- versus late-lesioned monkeys (10-fold difference). Increases in cell proliferation are suggested to last for only a few weeks after injury in rodents [44,56,57]. In non-human primates as well, the peak production of progenitor cells is around 9 days after damage [46,47]. The fact that the migration of immature neurons to the monkey amygdala is more than an order of magnitude greater following an early hippocampus lesion than following an adult lesion is likely due to a greater response of the neonatal brain to injury, which is characterized by a greater increase in cell proliferation, and a higher rate of migration and/or survival of neuroblasts [38,58]. The difference observed following early and adult hippocampus lesion could also suggest that the maturation and migration of neurons in the ventral amygdala is a mechanism of delayed postnatal plasticity in primates which could hardly be activated in adults [58].

### 3.4. Possible Functional Implications of Increased Neuronal Migration to the Amygdala

Although the influx and maturation of new neurons can be seen as an adaptive process to reduce the impact of brain damage [59], such massive changes in neuron number could also induce the disorganization of functional networks. Below, we discuss how the additional influx and maturation of a relatively large population of immature neurons in the amygdala following selective brain damage may alter amygdala connectivity and excitability, and ultimately be linked to behavioral alterations associated with certain neurodevelopmental disorders.

#### 3.4.1. Increase in Neuron Density

The increased density of mature neurons observed in the amygdala of lesioned monkeys implies that the neuropil, which contains glial elements, neuronal processes and synaptic contacts, is relatively reduced in these animals. Interestingly, the volume of the neuropil in the amygdala has been shown to become progressively larger from rats, to monkeys, to humans and is believed to reflect increasing connectivity [14]. Conversely, a reduction of the neuropil in human brains has been suggested as underlying altered neuroplasticity in neurodevelopmental disorders [60,61]. Accordingly, increases in mature neuron density observed in the paralaminar, lateral and basal nuclei could further impact amygdala functioning.

#### 3.4.2. Excitation-Inhibition Imbalance

The specific balance between excitation and inhibition could be altered in monkeys with lesions, which have increased neuron migration and maturation in the amygdala. Indeed, recently matured neurons in the developing primate amygdala have been shown to be primarily excitatory neurons [19]. Specifically, the majority of mature neurons in the paralaminar nucleus are excitatory neurons (TBR1 + VGLUT2+), and their number increases postnatally whereas the number of cells expressing interneuron markers (~3%) does not appear to change with age in the paralaminar nucleus [19]. An abnormal increase in the number of excitatory neurons in the amygdala, following hippocampal damage, for example, could impact the precise balance between excitation and inhibition, and lead to less efficient information processing [62]. Specifically, it has been shown that experimentally increasing the rate of neurogenesis in the adult dentate gyrus alters local circuit activity and excitability in rodents [63], and an increase in the number of amygdala excitatory neurons could similarly alter amygdala function. How these late-maturing neurons may be integrated into specific functional circuits remains to be investigated.

#### 3.4.3. Behavioral Alterations in Monkeys and Humans

The monkeys described here that received neonatal hippocampus lesions and exhibited associated evidence of post-lesion neuronal influx and maturation in the amygdala [13] were also involved in a series of studies investigating the neurobiological basis of affective processing and social behavior [27,64,65,66,67,68,69,70,71,72,73]. Interestingly, neonatal hippocampus lesions did not alter fundamental aspects of social behavior development between birth and two years of age. At these ages, hippocampus-lesioned monkeys behaved essentially like control animals with respect to the frequency and duration of a number of species-specific behaviors [27,64,65,68]. At four years of age, however, monkeys with early hippocampus damage were more social with their female peers than neurologically intact control monkeys and monkeys with neonatal amygdala lesions [72]. At seven years of age, monkeys with early hippocampus damage spent significantly more time engaging socially with their pair-mates than both controls and monkeys with neonatal amygdala lesions [71,73]. As an example, hippocampus-lesioned monkeys exhibited heightened communicative signaling and a propensity to engage in social interaction with their partners in an affiliative manner even after being aggressed [73].

Accordingly, early hippocampus damage induces changes in many brain systems that could, in turn, affect behavior. In the same group of monkeys with early hippocampus lesions, functional reorganization of surrounding cortical areas has been observed [74] and suggested to support some spatial learning and memory functions that are preserved following selective early [75] but not late [26] hippocampus lesions. In a human patient with developmental amnesia, early hippocampus damage was shown to impact the functional connectivity of many brain regions observed while the patient performed different memory tasks [76]. Thus, it may be difficult to entirely disentangle the differential impact of hippocampus damage, changes in amygdala neuron number and organization, and the concomitant reorganization of the functional connectivity with the rest of the brain with respect to behavioral changes observed in monkeys and humans with early hippocampus lesions. However, given the prominent role played by the amygdala in social cognition and emotional processing [77,78], it is reasonable to postulate that alterations of amygdala circuits may underlie abnormalities observed in these processes in neurodevelopmental syndromes such as autism spectrum disorders and Williams syndrome (WS).

The behavioral phenotype of WS, for example, is characterized by a hyper-affiliative social drive, marked by an atypically strong desire for social engagement, exaggerated gregariousness, a lack of inhibition in approaching and interacting with unfamiliar conspecifics, and impaired social perceptual ability [79]. Interestingly, a post-mortem stereological study found a greater number of neurons in the lateral nucleus of the amygdala in individuals with WS as compared to controls [80]. Moreover, we have recently shown major deficits in hippocampus-dependent spatial memory functions in individuals with WS [81,82], which are consistent with the structural and functional hippocampal abnormalities reported in individuals with WS [83,84]. It is thus interesting to consider the hypothesis, based on the behavioral and neuropathological findings obtained in monkeys, that abnormal hippocampus function may contribute to altered amygdala structure and function in individuals with WS, and thus impact social behavior and emotional processing in these individuals.

## 4. Conclusions

In recent years, a large population of immature neurons has been documented in the paralaminar nucleus of the primate amygdala. Here, we confirmed that the distribution of immature neurons extends to the anterior portions of the entorhinal cortex and layer II of the perirhinal cortex. We also provided novel arguments derived from stereological estimates of the number of mature and immature neurons, which support the view that the migration of immature neurons from the lateral ventricle accompanies neuronal maturation in the primate amygdala at all ages. We further showed that these processes were differentially impacted by selective hippocampus lesions performed at different ages. Altogether, our findings lead us to propose the hypothesis that increased migration and maturation of neurons in the amygdala following hippocampal dysfunction may be linked to behavioral alterations associated with certain neurodevelopmental disorders. Experimental studies in monkeys, with their phylogenetic proximity to humans, represent an unparalleled model in which systematic investigations of the normal and pathological development of brain-cognition interactions can be undertaken to test this hypothesis.

## 5. Materials and Methods

### 5.1. Experimental Animals

Forty-one macaque monkeys (*Macaca mulatta*) were used for this study. Monkeys were naturally born from multiparous mothers and raised at the California National Primate Research Center (CNPRC). Experimental procedures were approved by the Institutional Animal Care and Use Committee of the University of California, Davis, and were conducted in accordance with the National Institutes of Health guidelines for the use of animals in research. In order to reduce the number of animals used for research, all monkeys were involved in other studies, either before or after being recruited for this study.

#### 5.1.1. Unoperated Control Monkeys

Twenty-seven animals were used as controls: four 1-day-olds (2 males, 2 females), four 3-month-olds (2 M, 2 F), four 6-month-olds (2 M, 2 F), four 9-month-olds (2 M, 2 F), four 1-year-olds (2 M, 2 F) and seven adults (5.3–9.5 years old; 2 M, 5 F). These subjects were maternally reared in 2,000 m^2^ outdoor enclosures and lived in large social groups until they were killed. These animals were used in quantitative studies of the monkey hippocampal formation [38,85], entorhinal cortex [86,87] and amygdala [9,14].

#### 5.1.2. Adult Hippocampal-Lesioned Monkeys

Six animals (all males) were used in a study on the role of the hippocampus in spatial learning in adult macaque monkeys [26]. They were maternally reared in 2,000 m^2^ outdoor enclosures and lived in large social groups until about one year before experimental lesion surgery (at 6–9 years of age). At that time, each monkey was moved indoors and maintained in a standard home cage. They were 10.6–13.4 years of age at the time their brains were collected.

#### 5.1.3. Neonatal Hippocampal-Lesioned Monkeys

Eight animals (3 M, 5 F) were part of a longitudinal study of the effects of neonatal damage to the amygdala or hippocampus on the development of social behavior. Comprehensive rearing history has been described previously [64,71,75]. Briefly, infants were reared by their mothers in a socialization cohort consisting of six mother-infant pairs and one adult male. Infants were weaned from their mothers when the youngest member of each cohort reached six months of age. Infants were then permanently housed with their previously established cohort of six infants, one adult male and a new adult female. They were 9.1–9.4 years of age at the time their brains were collected.

#### 5.1.4. Experimental Lesion Surgeries

Bilateral hippocampus lesions were performed following the same protocol for both neonatal (12–16 days after birth) and adult (at 6.7–9.7 years of age) lesion groups. Detailed procedures are described in [26,64].

### 5.2. Histological Procedures

#### 5.2.1. Brain Acquisition

Monkeys were deeply anesthetized with an intravenous injection of sodium pentobarbital (50 mg/kg; Fatal-Plus, Vortech Pharmaceuticals, Dearborn, MI) and perfused transcardially with 1% and then 4% paraformaldehyde in 0.1 M phosphate buffer (PB; pH 7.4) following standard protocols [88].

#### 5.2.2. Nissl Staining

One series of 30- or 60-μm sections were collected in 10% formaldehyde solution in 0.1 M PB (pH 7.4) and postfixed at 4 °C for 4 weeks prior to Nissl staining. Other series were collected in tissue collection solution and kept at −70 °C until further processing. The procedure for Nissl-stained sections followed our standard laboratory protocol [88].

#### 5.2.3. Neuronal Phenotype Markers

We used two cell specific markers, NeuN and Bcl2, to characterize the phenotypes of neurons in the medial temporal lobe. NeuN is a well-established neuron-specific marker [89]. Bcl2 is an anti-apoptotic protein, which influences the rate of neuronal differentiation in young neurons and is expressed in populations of immature neurons [2]. Thirty-µM thick sections were rinsed 3 × 10 min in 0.1 M phosphate-buffered saline (PBS, pH 7.4), and incubated for 96 h in primary antiserum at 4 °C with gentle agitation on a rotating platform (1:50 mouse anti-Bcl2 (Abcam, Cambridge, UK; Ab694) + 1:1000 guinea-pig anti-NeuN (Merck Millipore, Billerica, MA, USA; ABN90P) in 0.1 M PBS + 0.1% Triton X−100 + 10% bovine serum (Pan Biotech, Aidenbach, Germany; P30–0602)). Sections were then rinsed 10 min in 0.1 M PBS, 2 × 10 min in Tris buffer and incubated for 4 h at room temperature in secondary antiserum with gentle agitation on a rotating platform (1:400 Alexa Fluor^®^ 488-conjugated goat anti-mouse IgG (Thermo Fisher Scientific, Waltham, MA, USA; A-11029) + 1:400 Alexa Fluor^®^ 568-conjugated goat anti-guinea-pig IgG (Thermo Fisher Scientific, Waltham, MA, USA; A-11075) in Tris buffer). From this point on, sections were protected from light. Sections were rinsed 10 min in Tris buffer, 2 × 10 min in 0.1 M PBS, mounted on gelatin-coated slides and air-dried overnight at room temperature. Sections were dehydrated through a graded series of ethanol solutions and coverslipped with DPX new (Merck KGaA, Darmstadt, Germany; 100579).

### 5.3. Data Acquisition and Statistical Analyses

#### 5.3.1. Stereological Analyses on Nissl-Stained Sections

We used the optical fractionator method [90] on Nissl-stained sections to estimate the number of mature and immature neurons in the paralaminar, lateral and basal nuclei of the amygdala (Table 1 and Table S1). Neuron number was estimated in the right or left amygdala, as determined pseudo-randomly for each individual. We used a 100× Plan Fluor oil objective (N.A. 1.30) on a Nikon Eclipse 80i microscope (Nikon Instruments, Melville, NY) linked to PC-based StereoInvestigator 11.0 (MBF Bioscience, Williston, VT, USA). Section thickness was measured at every other counting site. We distinguished mature and immature neurons from other cells, based on morphological criteria identifiable in Nissl preparations [9,13,38]. Briefly, neurons are darkly stained and comprise a single large nucleolus. Immature neurons are small with round to slightly oval, hyperchromatic nuclei containing distinguishable nucleoli [3,4,5]. We estimated the volume of the amygdala nuclei (lateral, basal, and paralaminar) according to the Cavalieri principle on Nissl-stained sections [90,91].

#### 5.3.2. Neuronal Phenotypes

We used a confocal microscope (TCS SP5, DM6000 CFS, Leica Microsystems, Wetzlar, Germany) to determine the phenotype of immature neurons in the medial temporal lobe, based on the co-localization of Bcl2 and NeuN (Figure 2). We used a 40× objective corrected for chromatic and spherical aberration (HC PL APO 40×/1.30 oil CS2) and performed a sequential acquisition to avoid ‘cross-talk’ between different excitation lasers and photomultiplier detection systems. Bcl2 visualization (fluorophore Alexa Fluor^®^ 488): excitation: 488 nm (argon laser); detection: 505–539 nm, gain 711, offset −1.2, pinhole 1.93 AU/126 μm. NeuN visualization (fluorophore Alexa Fluor^®^ 568): excitation: 561 nm (DPSS laser); detection: 585–624 nm, gain 805, offset 0.7, pinhole 1.93 AU/126 μm. For each acquisition, we used z-steps of 0.5 μm. We analyzed Bcl2-positive cells using Fiji/ImageJ software version 1.50b (NIH, Bethesda, MD, USA).

## Figures and Tables

**Table 1:** Neuron numbers in three anteroposterior segments of the paralaminar nucleus of the amygdala in control rhesus monkeys at different postnatal ages (mean ± SD).

**Immature neurons**				
	0–3-month	6–9-month	1-year	Adult
Anterior	320,116 ± 52,162	365,778 ± 72,720	278,854 ± 34,880	92,609 ± 22,334
Middle	447,066 ± 117,968	429,655 ± 84,484	349,521 ± 21,913	168,363 ± 41,566
Posterior	275,609 ± 139,239	217,477 ± 68,508	264,992 ± 67,537	237,238 ± 69,921
TOTAL	1,042,791 ± 250,175	1,012,910 ± 193,789	893,367 ± 71,509	498,210 ± 82,884
**Mature neurons**				
	0–3-month	6–9-month	1-year	Adult
Anterior	23,708 ± 5,900	21,473 ± 14,113	25,880 ± 4,387	127,076 ± 26,604
Middle	28,775 ± 9,750	20,744 ± 13,227	20,660 ± 6,018	186,252 ± 30,750
Posterior	15,990 ± 13,555	12,178 ± 12,696	14,100 ± 6,516	93,796 ± 21,515
TOTAL	68,473 ± 14,508	54,395 ± 31,898	60,640 ± 5,811	407,123 ± 37,270

## Data Availability

All data that are not published are available in the Supplementary Materials.

## Supplementary Materials

The following are available online at https://www.mdpi.com/article/10.3390/ijms22136691/s1, Table S1: Neuron numbers and volumes of the main amygdala nuclei in rhesus monkeys: juvenile controls, adult controls, and adults subjected to early or late hippocampus lesion, Figure S1: Anteroposterior distributions and numbers of immature neurons and Bcl2-positive cells in the paralaminar nucleus of the adult monkey.
