# Supplementary material for: Life and Death of Immature Neurons in the Juvenile and Adult Primate Amygdala
Source: Int J Mol Sci. 2021 Jun 22;22(13):6691. doi: 10.3390/ijms22136691 (PMC8268704; doi:10.3390/ijms22136691)
Supplement: Supplementary file 1 [file ijms-22-06691-s001.zip › ijms-1235970-supplementary.pdf]

International Journal of *Molecular Sciences*

MDPI

Table S1. Neuron numbers and volumes of the main amygdala nuclei in rhesus monkeys: juvenile controls, adult controls, and adults subjected to early or late hippocampus lesion (mean ± SD).

	**Immature neurons**	**Mature neurons**			**Volume (mm³)**		
	Paralaminar	Paralaminar	Lateral	Basal	Paralaminar	Lateral	Basal
Juvenile control (*n* = 20)	1,000,953 ± 202,326	61,275 ± 22,351	1,742,168 ± 249,743	1,286,503 ± 143,988	4.0 ± 1.3	32.3 ± 5.1	34.3 ± 6.7
Adult control (*n* = 7)	498,210 ± 82,884	407,123 ± 37,270	1,706,444 ± 178,120	1,573,016 ± 212,939	7.9 ± 0.8	35.6 ± 5.0	43.7 ± 5.2
Adult late-lesioned (*n* = 6)	364,684 ± 91,012	670,142 ± 69,897	1,847,354 ± 340,830	1,470,181 ± 353,532	10.0 ± 0.9	37.9 ± 6.2	44.1 ± 8.9
Adult early-lesioned (*n* = 8)	686,313 ± 85,446	700,264 ± 27,369	2,454,814 ± 247,625	2,125,273 ± 395,511	9.9 ± 0.7	38.6 ± 5.4	48.2 ± 7.6

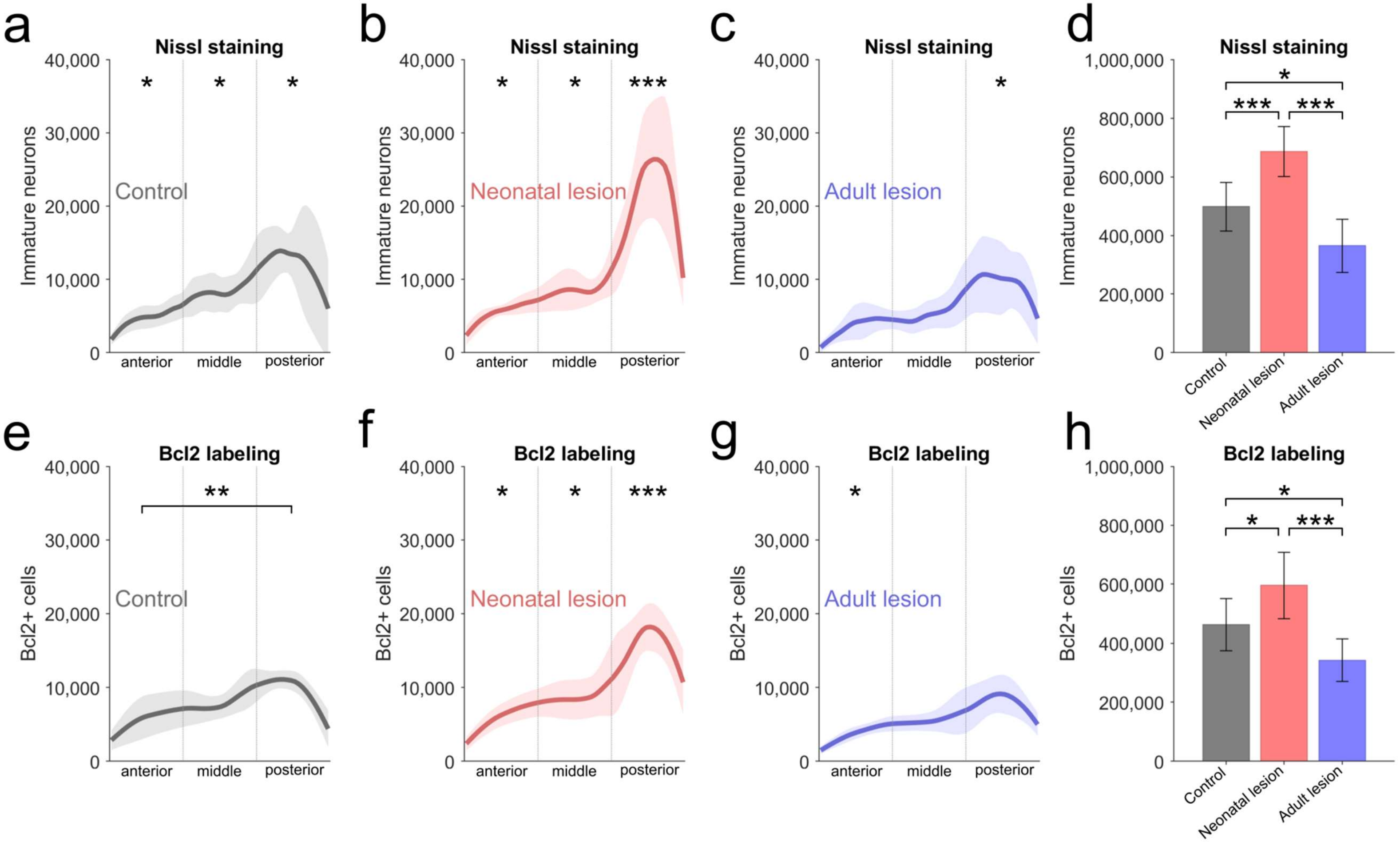

Figure S1. Anteroposterior distributions and numbers of immature neurons and Bcl2+ cells in the paralaminar nucleus of the adult monkey. Stereological estimates of the number of immature neurons based on Nissl-stained (**a**–**d**) or Bcl2-immunolabeled (**e**–**h**) brain sections. Unoperated control ($n = 7$; grey; **a**, **e**), neonatal hippocampus-lesioned ($n = 8$; red; **b**, **f**), and adult hippocampus-lesioned ($n = 6$; blue; **c**, **g**). Comparison of the number of cells between the three anteroposterior segments (**a**–**c**, **e**–**g**) (asterisk positioned above a single segment means that this set of values is significantly different from the other two segments). Comparison of the number of cells between experimental groups (**d**, **h**). Average ± SD; *$p < 0.05$; **$p < 0.01$; ***$p < 0.001$.
